# Linking management planning for coastal wetlands to potential future wave attenuation under a range of relative sea-level rise scenarios

**DOI:** 10.1371/journal.pone.0216695

**Published:** 2019-05-14

**Authors:** Ann Commagere Hijuelos, Jasper T. Dijkstra, Tim J. B. Carruthers, Karel Heynert, Denise J. Reed, Bregje K. van Wesenbeeck

**Affiliations:** 1 The Water Institute of the Gulf, Baton Rouge, LA, United States of America; 2 Deltares, Marine and Coastal Systems, Delft, The Netherlands; University of Sydney, AUSTRALIA

## Abstract

Understanding changes in wave attenuation by emergent vegetation as wetlands degrade or accrete over time is crucial for incorporation of wetlands into holistic coastal risk management. Linked SLAMM and XBeach models were used to investigate potential future changes in wave attenuation over a 50-year period in a degrading, subtropical wetland and a prograding, temperate wetland. These contrasting systems also have differing management contexts and were contrasted to demonstrate how the linked models can provide management-relevant insights. Morphological development of wetlands for different scenarios of sea-level rise and accretion was simulated with SLAMM and then coupled with different vegetation characteristics to predict the influence on future wave attenuation using XBeach. The geomorphological context, subsidence, and accretion resulted in large predicted reductions in the extent of vegetated land (e.g., wetland) and changes in wave height reduction potential across the wetland. These were exacerbated by increases in sea-level from +0.217 m to +0.386 m over a 50-year period, especially at the lowest accretion rates in the degrading wetland. Mangrove vegetation increased wave attenuation within the degrading, subtropical, saline wetland, while grazing reduced wave attenuation in the temperate, prograding wetland. Coastal management decisions and actions, related to coastal vegetation type and structure, have the potential to change future wave attenuation at a spatial scale relevant to coastal protection planning. Therefore, a coastal management approach that includes disaster risk reduction, biodiversity, and climate change, can be informed by coastal modeling tools, such as those demonstrated here for two contrasting case studies.

## Introduction

There has been increasing global recognition among coastal managers of the importance of conserving or restoring vegetation to increase coastal resilience through ecosystem service provision, such as the attenuation of waves to reduce coastal flooding and marginal erosion [1–6]. Quantification of this functionality of wetlands is demonstrated by multiple studies that present detailed measurements of wave attenuation by coastal vegetation communities [4,7–10] with some showing upwards of 50% wave reduction within the first ten meters of vegetation. Although these measurements demonstrate the role that wetlands potentially play in reducing impacts of waves and surge [11–13], the provision of ecosystem services is recognized to be non-linear in space and time [14,15]. As wetland ecosystems decline globally [16] due to multiple pressures, such as clear cutting for aquaculture [17], nutrient input [18], and changing sediment input [19] and respond to future sea-level rise [20–23], the extent to which wetlands will continue to perform this role under future conditions is uncertain. Understanding potential change in realized wave attenuation over time will be crucial to the incorporation of coastal wetlands into holistic coastal risk management [24,25].

Restoration and management policies and approaches, such as the Coastal Wetlands Planning, Protection and Restoration Act (Public Law 101–646, Title III), in the United States, or the Habitat Directive (Council Directive 92/43/EEC), in the European Union, often focus on maintaining wetland structure, e.g., the extent and type of vegetated wetlands. This approach relies on the assumption that functions such as wave attenuation will be achievable whenever some vegetation structure is present, but the diverse and complex structure of coastal wetlands means that the provision of specific services among locations or over time, can vary greatly [26]. Numerical tools that could allow resource managers to benefit from rigorous application of knowledge about wave attenuation by coastal vegetation into their decision making are available [27,28], although they have not been widely used to quantify changes in wave attenuation over time as sea-level rise and sediment accretion alter wetland extent and configuration. Similarly, models that predict future wetland extent under various sea-level rise scenarios [29,30] may not provide adequate insight on potential changes in key ecosystem functions, such as wave attenuation. The objective here is to demonstrate how integrating models to predict changes both in wetland extent and subsequent wave attenuation potential can be of support to management decisions. We utilized the Sea Level Affecting Marshes Model (SLAMM) [31,32] and XBeach [33–35] model to assess changes in wetland wave attenuation over time in response to sediment accretion, sea-level rise, management actions, and their interactions. Managers often need simple tools to explore a range of possibilities prior to more detailed analysis [36]. SLAMM has been used in many coastal areas to assess future vulnerability to sea-level rise [37,38] and more recently it has been used to explore cost-benefit of management strategies from stakeholder-generated values of ecosystem services [39]. While several detailed models are available for simulations of wave propagation e.g., [40,41], XBeach has been validated for a series of flume and field experiments including vegetation [33,42,43] and can be applied relatively simply to cross shore two-dimensional analysis. Such an approach could be used to support resource managers and decision makers in disaster risk reduction and adaptation [44,45]. Here the models are applied to two contrasting coastal wetlands providing case studies not only of the application of the integrated models, but also of the utility of the results in assessing wave attenuation by coastal vegetation in two areas where coastal wetlands form a vital part of coastal defense measures: coastal Louisiana and the Dutch Wadden Sea.

## Methods

### Study areas

**Fig 1:**
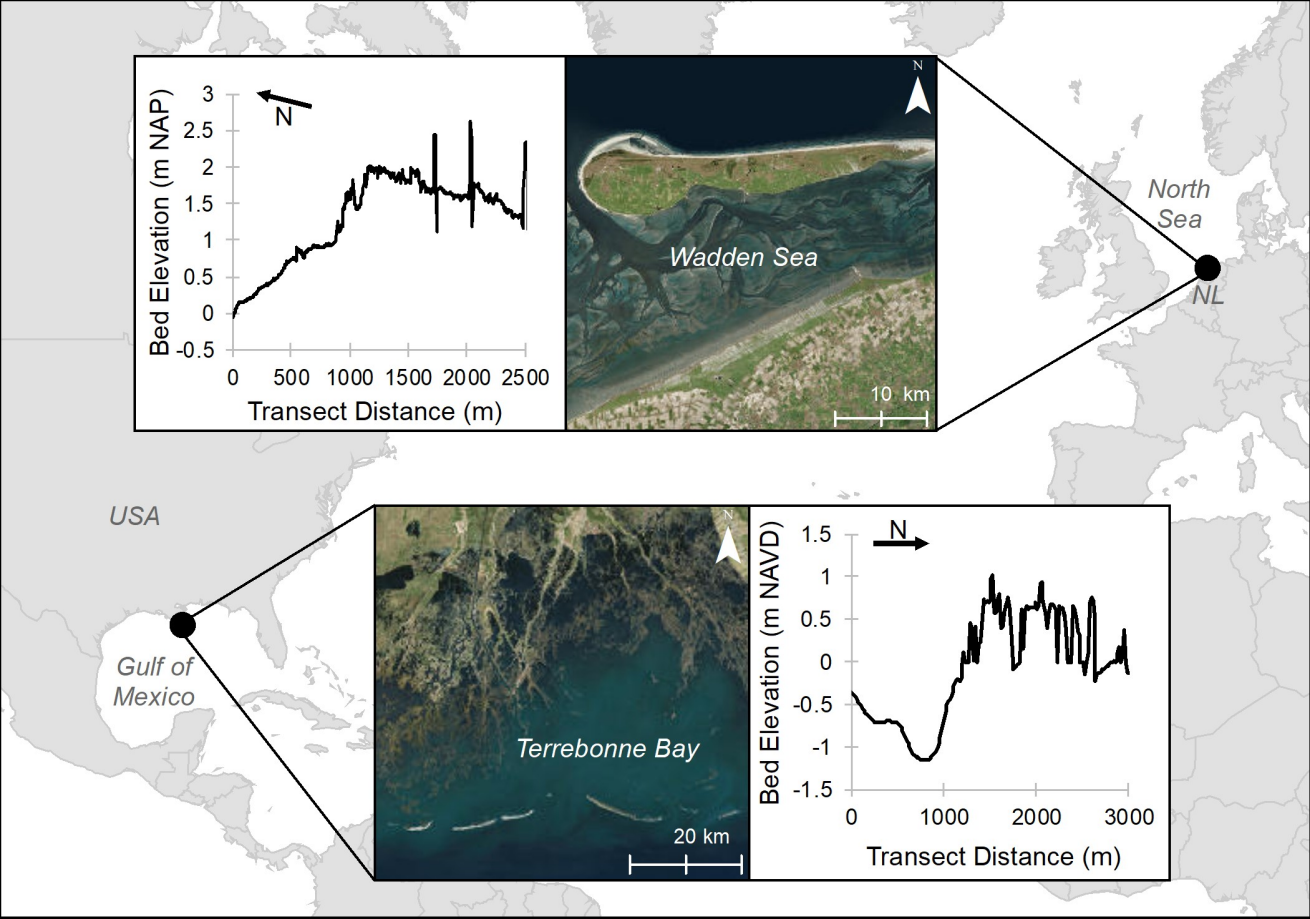
General location of sites selected to illustrate contrasting coastal wetlands: A prograding system with plentiful sediment supply (Friesland, Wadden Sea, the Netherlands) and a degrading system with limited sediment supply (Mississippi Delta, LA, USA). Arrow refers to North direction. Created with ESRI ArcGIS 10.6.1 software. Basemap satellite images accessed from World Imagery ESRI Tile Layer located in: https://services.arcgisonline.com/ArcGIS/rest/services/World_Imagery/MapServer (credits: Esri, DigitalGlobe, GeoEye, Earthstar Geographics, CNES/Airbus DS, USDA, USGS, AeroGRID, IGN, and the GIS User Community). Background basemap accessed from World Countries (Generalized) ESRI Feature Layer located in https://services.arcgis.com/P3ePLMYs2RVChkJx/arcgis/rest/services/World_Countries_(Generalized)/FeatureServer (credits: Esri, DeLorme Publishing Company, Inc.).

The two case studies are both in areas where coastal wetlands exist on the seaward side of earthen defense levees and where levee management is considered essential for the future of coastal communities and livelihoods. These case studies also illustrate the range of conditions experienced by coastal wetlands: a degrading system with limited sediment supply and considerable subsidence (Mississippi Delta, LA, USA) and a prograding system with plentiful sediment supply and negligible subsidence (Friesland, Wadden Sea, the Netherlands [NL]) (Fig 1). Both areas have undergone extensive changes over the last several decades and climate change and management actions are expected to influence the future elevation, area, or species composition of wetlands [46–51]. Wave attenuation and its potential impact on wave runup is of key interest in both of these areas, and management options related to the construction or preservation of vegetation in front of coastal protection levees are being explored [44,52,53]. The role of wetlands considered in management plans may be best evaluated if their future effectiveness under a range of different future scenarios can be quantified. Thus, these areas can serve as exemplary study sites for investigating the effect of future coastal wetland changes on risk management.

In the Mississippi Delta, natural forces such as subsidence and hurricanes have combined with river management and hydrologic alteration to produce rapid wetland loss [54,55]. The value of wetland extent and character has been directly related to damage avoidance of private property resulting from storm surges [56]. Some urban areas are surrounded by extensive protection infrastructures, (e.g., New Orleans, Louisiana, USA) but many coastal communities remain unprotected. Thus, an approximately 150 km series of levees and floodgates is being planned to provide protection from storm surges. This Morganza to the Gulf project [57] largely follows existing areas of higher land along natural bayous but there are several locations where it crosses coastal wetlands and these levee sections could potentially benefit from wave attenuation by surrounding wetlands, or conversely be vulnerable to erosion from waves if the current outboard wetlands further degrade. The vegetation in the selected Louisiana site (Fig 1) is currently dominated by *Spartina patens*. However, native black mangroves, *Avicennia germinans*, are also found approximately 15 km south of the proposed levees site and have the potential to expand northerly with predicted reductions in freezing temperatures [58]. As a result, planting mangroves could serve as a cost-effective means for local managers to protect levee systems from erosion caused by waves [59].

In the Wadden Sea, sedimentation fields protected by brushwood groynes have historically been used to stimulate accretion and create wetlands subsequently reclaimed for agricultural uses [60]. As the socioeconomic condition of the region evolved in the mid-twentieth century, the reclamation of wetlands for agriculture was reduced and the natural value of wetlands was recognized [61,62]. New sedimentation fields are not currently promoted, as they reduce the area of intertidal flats, which are protected habitats themselves and valued ecosystems [63]. The limited intertidal area backed by the levees and a plentiful sediment supply results in a narrow transition from intertidal flats to constructed wetlands [62,63]. As a result, most foreland wetlands lack pioneer species and are predominantly vegetated by climax species such as *Atriplex portulacoides* on the low wetland and *Elytrigia atherica* on the high wetland [64]. Strategic management actions, such as livestock grazing with varying intensities, have been used to increase biodiversity in these wetland assemblages [63]. The possible protective role of wetlands and their vegetation is not part of the present legal procedure for levee safety evaluation because the long-term presence and management of the wetlands is considered uncertain. A more advanced evaluation procedure that will be applied in the near future, however will likely necessitate costly dike reinforcements [65]. The result of using wetlands as part of the coastal defence is under re-consideration and therefore understanding the influence of grazing on the ability of wetlands to attenuate waves is an important consideration.

### Model application

Two linked models were used to evaluate the interactive effects of future climate change and management decisions on potential wave attenuation over a coast-normal transect over a 50-year period, in line with common levee safety evaluation procedures. Within each site, a transect was selected with initial elevations and wetland-water configuration representative of conditions found within the respective larger regions (Fig 1; Table 1). Levee safety assessments [66] are based on an evaluation of stretches of levees (typically 100’s to 1000 m long) with fairly uniform hydraulic loads and geotechnical conditions. The reduction of nearshore waves at the toe of the levee required for levee design is determined over a transect perpendicular to the levee. While this ignores longshore gradients, it is computationally efficient and provides an effective tool for use in exploring management options.

**Table 1:** Parameters and their associated values used in model setup and scenario runs.

Model	Parameters	Louisiana	Netherlands
**SLAMM**	**Bed Elevation**	-1.16 to 1.02 m NAVD88 (Digital elevation model obtained from [72])	-0.06 to 2.62 m NAP (Digital elevation model obtained from [73])
	**Land Use Land Cover**	LULC map obtained from [72]	LULC map obtained from [74]
	**Sea-level rise**	21.7, 31.9, 38.6 cm by 2060 relative to 1990 levels [75]	21.7, 31.9, 38.6 cm by 2060 relative to 1990 levels [75]
	**Accretion**	5.7, 9.1, 12.5 mm yr^-1^ [76]	1.3, 20.0, 56.3 mm yr^-1^ [77] (includes subsidence)
	**Subsidence**	8.8 mm yr^-1^ [78]	N/A
**XBeach**	**Return Period**	1/100 [57]	1/4000 [79]
	**Significant wave height**	1.86 m [57]	1.85 m [79]
	**Peak period**	7.1 s [57]	6.3 s [79]
	**Surge level**	4.36 m NAVD88 [57]	4.9 m NAP [79]
	**Species**	Herbaceous: *Spartina patens* Mangrove: *Avicennia germinans*	*Salicornia spp*.
	**Drag coefficient (C**_**D**_**)**	Herbaceous: 0.18 [10] Mangrove: 1.0 [80]	0.19 [10]
	**Vegetation Layers**	Herbaceous: 1 layer Mangrove: 6 layers	1 layer
	**Vegetation Height**	Herbaceous: 1.072 m [81] Mangrove: 0.2, 0.5, 1.0, 1.5, 2.0, 2.5 m *	Base: 0.3 m [82] Grazing: 0.05 m [83]
	**Stem Width**	Herbaceous: 0.0022 m [81] Mangrove: 0.0086, 0.06, 0.035, 0.035, 0.015, 0.008 m *	0.00125 m
	**Stem Density**	Herbaceous: 629 m^-2^ [81] Mangrove: 180, 0.6, 2.7, 1.5, 1, 0.5 m^-2^ *	1225 m^-2^

* indicates data were estimated for this study

The models selected are in current use for independently assessing management options [67,68] either to assess wetland response to sea-level rise, or to examine wave attenuation. The purpose of linking them is to demonstrate that existing models can be used together to provide greater insight than they can alone. SLAMM (version 6.2) was used to simulate the effects of accelerated sea-level rise rates and accretion on the presence and absence of wetland habitats. SLAMM is open source software that has been widely used in coastal areas globally to investigate future sustainability of wetland habitats, and many coastal managers are familiar with its application providing readily available model comparisons and well-documented model capabilities and limitations [38,69–71]. The model simulates the dominant processes in wetland development by using a decision-tree approach to convert one habitat type to another based on elevation, slope, accretion, subsidence, erosion, and habitat type [32]. Previous applications of SLAMM to coastal Louisiana illustrated through hindcasting simulations that SLAMM was accurate in its predictions of saline wetland loss [71]. Inputs to the cell-based model included bed elevation and land use land cover data, subsidence, and accretion, specific to the study area and global sea-level rise rates (Table 1). The model was run at 15 m horizontal resolution for the Louisiana site and 5 m for the Netherlands study site, based on the digital elevation model resolution of the input data for both systems (Table 1).

Three sea-level rise scenarios were selected from the limited set of options provided in the SLAMM interface at the time of the study and applied to both study sites (Table 1). Although these levels are now considered to be at the conservative end of recent predictions of future sea-level rise [84], the various rates provide a range of potential future change. Managers will need to consider the implications of different sea-level rise scenarios as part of their decision-making process. Further, given uncertainty regarding future accretion rates and the sensitivity of the model to this parameter [38,71], three accretion rates for each study area were used. Parameterization of the accretion variable for Louisiana was derived from the mean and one standard deviation of long-term accretion rates (1963–2006) measured in Cesium cores taken within the region [76] (Table 1). The accretion variable for the Netherlands was derived from observed elevation changes between 2001 and 2009. The accretion rate varies considerably over the elevation range of the marsh, so an elevation related accretion curve was used [20], to generate the lowest, median and highest percentile values for the accretion rate. As these are observed accretion rates, they include effects of vegetation on sedimentation, compaction, subsidence and average out temporal variability induced by storms during the period they represent [77]. The difference in methods used for accretion between the sites reflect different periods and process histories However, these are the types of information often available to managers, and the use of three values at each site provides a broad view of how accretion could vary in the future. The assumption is also made that change in elevation is equal to that of the accretion rate. Studies in Louisiana show that accretion may not always result in an equivalent increase in elevation due to shallow subsidence (e.g., [85]) but differences are also site specific. As this study is not focused on actual predictions of change in any one wetland, but exploration of how changes in sea-level rise and accretion interact with management actions to influence wave attenuation, this assumption is considered reasonable.

A 50-year time horizon was selected to reflect the current management planning in both Louisiana and the Netherlands [52,66]. A total of nine simulations each characterized by combinations of three sea-level rise rates and three accretion rates were run in SLAMM over a 50-year period to produce output of bed elevation and presence and absence of wetland vegetation across the transect.

The output from SLAMM (i.e., bed elevation and wetland presence/absence along a 3000 m by 15 m transect for LA site, 3000 m by 5 m transect for Netherlands site) was then used in XBeach (version 1.22.4672) [42] to model the effect of wetland presence and vegetation resistance on wave attenuation under storm conditions. XBeach is an open source model for wave propagation, mean flow, sediment transport and morphological changes of the nearshore area and has been validated for a series of flume and field experiments including vegetation [33,42,43]. XBeach uses an expression for time-averaged energy dissipation due to vegetation that Mendez & Losada (2004) derived for random waves over an arbitrary bed [35]. Energy dissipation by vegetation is based on properties that may vary with plant height, such as stem diameter and density, as well as a bulk drag coefficient (C_D_), which enables a more realistic representation of different types of vegetation than in other wave models that account for vegetation, e.g. Simulating Waves Nearshore (SWAN) [86]. Our model integration focused on representing how the morphological and vegetation changes altered wave attenuation. This application did not consider feedbacks between wave attenuation and accretion [87] which would be an important future development but likely entails more complex analyses than those used here.

### Representative storm conditions and vegetation properties

The design storm conditions for levees differ considerably between Louisiana and the Netherlands [88]. Louisiana’s hurricane risk reduction levees are normally designed to provide protection from water levels with a return period of 1 in 100 years [57]. The design conditions for the levee adjacent to the study site are a water level of 4.36 m NAVD 88, a significant wave height of 1.86 m and a peak period of 7.1 s. The levee safety evaluation procedure in the Netherlands has previously been based on the economic risk of flooding the hinterland with a return period of 1 in 4000 years for this part of the country [66], although more complex procedures have been recently implemented [89]. The design conditions corresponding to the simpler 1 in 4000 years return period are a water level of 4.9 m above MSL, a significant wave height of 1.85 m and a peak period of 6.2 s for the area of interest [79,88]. The design storm conditions used in this study include relatively short wave periods compared to those considered in other studies of tropical mangrove systems [9,90] that noted more limited wave attenuation for longer period waves. However, these studies found that mangroves were effective at attenuating short period waves (5–6 seconds). Furthermore, using the design storm conditions in this analysis shows how model output results can be relevant to current planning frameworks for disaster risk reduction in the systems studied and demonstrates the utility of the approach for systems subject to varied storm conditions.

XBeach requires parameterization of the vegetation characteristics and bulk drag coefficient (C_D_), which is related to the interplay between hydrodynamics and marsh vegetation properties such as size, flexibility and relative submergence. Base vegetation characteristics were parameterized using knowledge of locally occurring plant species. In coastal Louisiana, vegetation characteristics were parameterized in the model using an average of localized measurements of *Spartina patens* collected near the site of interest (Table 1). Plant characteristics are predominately uniform across the vertical profile of *Spartina patens*. As a result, one set of characteristics was used to represent the entire stem. The vegetation was parameterized with a vegetation height of 1.07 m, a stem diameter of 2.2 mm and 629 stems per m^2^ [81]. The exponential decay relationship between stem Reynolds number and C_D_ used in previous studies [10,27] and the vegetation characteristics parameterized in the model, were used to derive the drag coefficient (C_D_ = 0.18).

In the Dutch Wadden Sea area under investigation, the wetlands are characterized by a relatively narrow band of pioneer species on the seaward side (*Salicornia procumbens*, *Spartina anglica*) and a large area of established wetland to the landward side, covered with a mixed canopy of *Elymus athericus*, *Puccinellia maritima* and *Elytigia atherica* typical of mid to high wetland communities in northwest Europe. This vegetation composition is very similar to the vegetation in the large flume experiment on wave attenuation by saline wetlands under storm conditions [10]. The vegetation was parameterized with a vegetation height of 30 cm, a stem diameter of 1.25 mm and 1225 stems per m^2^. As was used for the LA study area, previous studies [10,27] provided the relation between C_D_ and Reynolds number for these flexible plant species that were used to derive the drag coefficient (C_D_ = 0.19) [27].

In addition to exploring changes in wave attenuation over time due to sea-level rise and accretion in degrading and prograding systems, analysis was conducted to explore the effects of intrinsic (local) management actions on wave attenuation under storm conditions. For degrading Louisiana wetlands, expansion of native *A*. *germinans*, is predicted to occur because of reductions in freezing temperatures [58]. As a result, planting mangroves could serve as a means for local managers to ensure wetlands continue to provide protection to levees, or to enhance the protection compared to current herbaceous species. To test the potential wave attenuation effect of mangroves compared to herbaceous wetland, XBeach was parameterized with vegetation characteristics representative of mature *A*. *germinans*, found within coastal Louisiana (Table 1). In XBeach, energy dissipation by vegetation is based on measurable vegetation properties that may vary over the height of the plant. As a result, vegetation can be parameterized across a number of vertical layers that represent the specific properties of vegetation at different heights. For this aspect of the study XBeach was parameterized with 6 vertical vegetation layers, representative of mature *A*. *germinans*, found within coastal Louisiana: 0–20, 20–50, 50–100, 100–150, 150–200 and 200–250 cm (Table 1). Each layer represents the characteristics of the plant at that height above the ground. To parameterize each vegetation layer in XBeach, number of stems and pneumatophores (entirely within the 0–20 cm layer), height, and diameter were estimated for mature *A*. *germinans* trees from field photographs taken near Grande Isle, Louisiana (Table 1). These estimated values per tree were then converted to m^2^ assuming 100 trees per hectare as derived from Osland et al. 2012 [91] for a created mangrove wetland. Although no direct measurements of C_D_ within mangrove stands under storm conditions were available in the literature at the time of this study, C_D_ = 1 has previously been used to represent rigid vegetation in experimental studies [92–94], characteristic of mangrove roots and trunks. Thus, a bulk drag coefficient of 1.0 was used to parameterize the model.

In the Dutch Wadden Sea, the wetlands are partly used as pasture for livestock and grazing to promote species diversity, as previously described [95]. The effect of this management was incorporated in the model by reducing the vegetation height to 5 cm whilst keeping the density and stem diameter constant [83].

### Analysis of model outputs

For each of the nine future change simulations (three SLR and three sediment accretion rates) over the two study sites, SLAMM outputs included presence and absence of wetland vegetation. We then calculated the percent of the modelled transect that had vegetation present, referred to as ‘wetland extent’, and evaluated how wetland extent changed over the 50-year simulation period under the nine scenarios. From the XBeach output, we extracted wave heights at the beginning of the transect and end of the transect and calculated wave attenuation as the difference between the two. Higher wave attenuation values indicate smaller waves present at the end of the transect. To separate the specific effect of vegetation on wave attenuation, the difference in wave attenuation between simulations including vegetation and simulations where vegetation was purposefully excluded (i.e., bare ground) was calculated.

## Results

### Effects of SLR, sediment accretion, and vegetation cover on future wave attenuation

**Fig 2:**
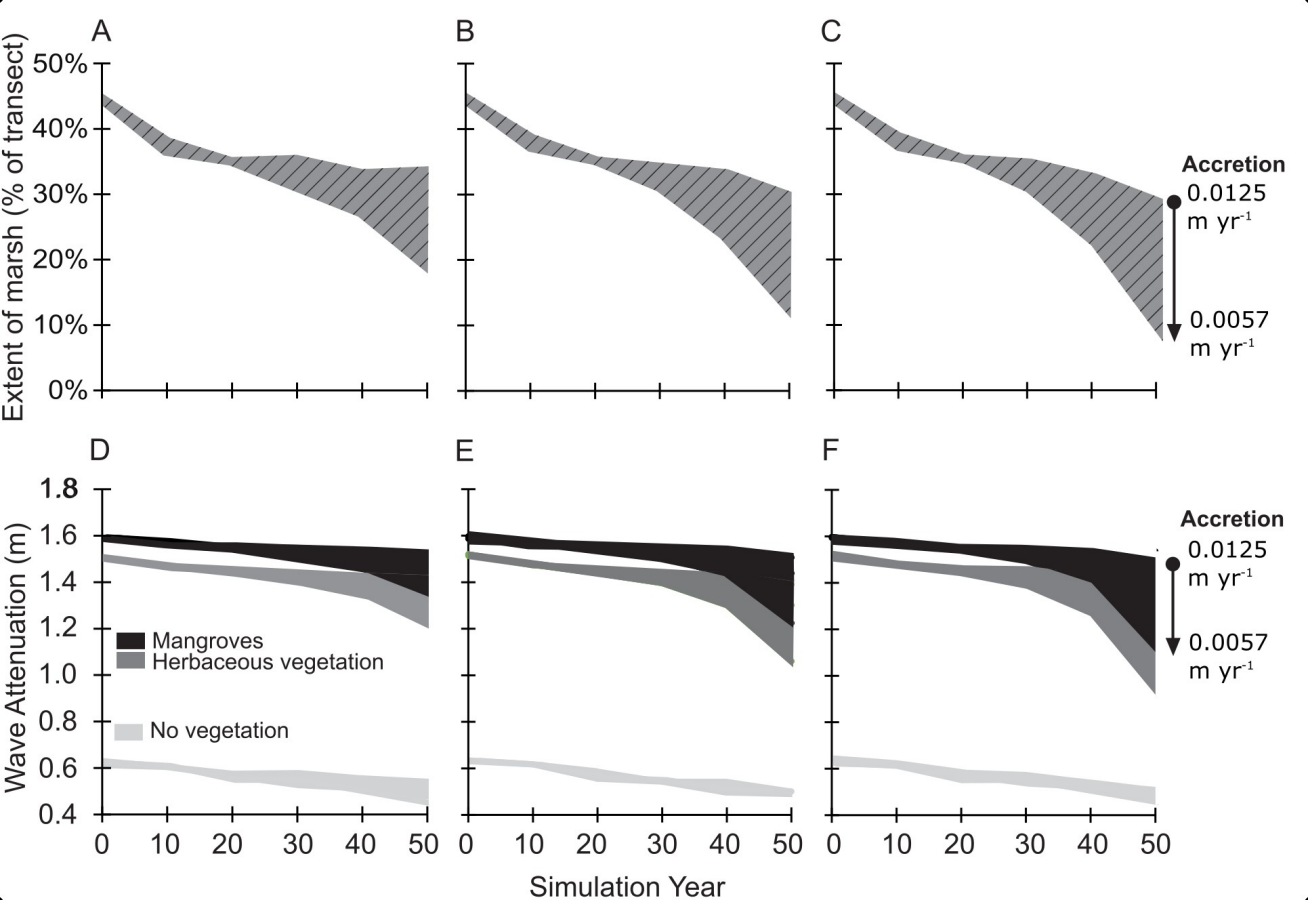
Change in wetland extent and wave attenuation in a degrading wetland (Mississippi Delta) under a range of sea-level rise and accretion scenarios and management actions. Extent of marsh and wave attenuation reported as a range across the three accretion rates (Table 1) and for three sea level rise scenarios: (A,D) 0.217 m of SLR, (B,E) 0.319 m of SLR, and (C,F) 0.386 m of SLR.

**Fig 3:**
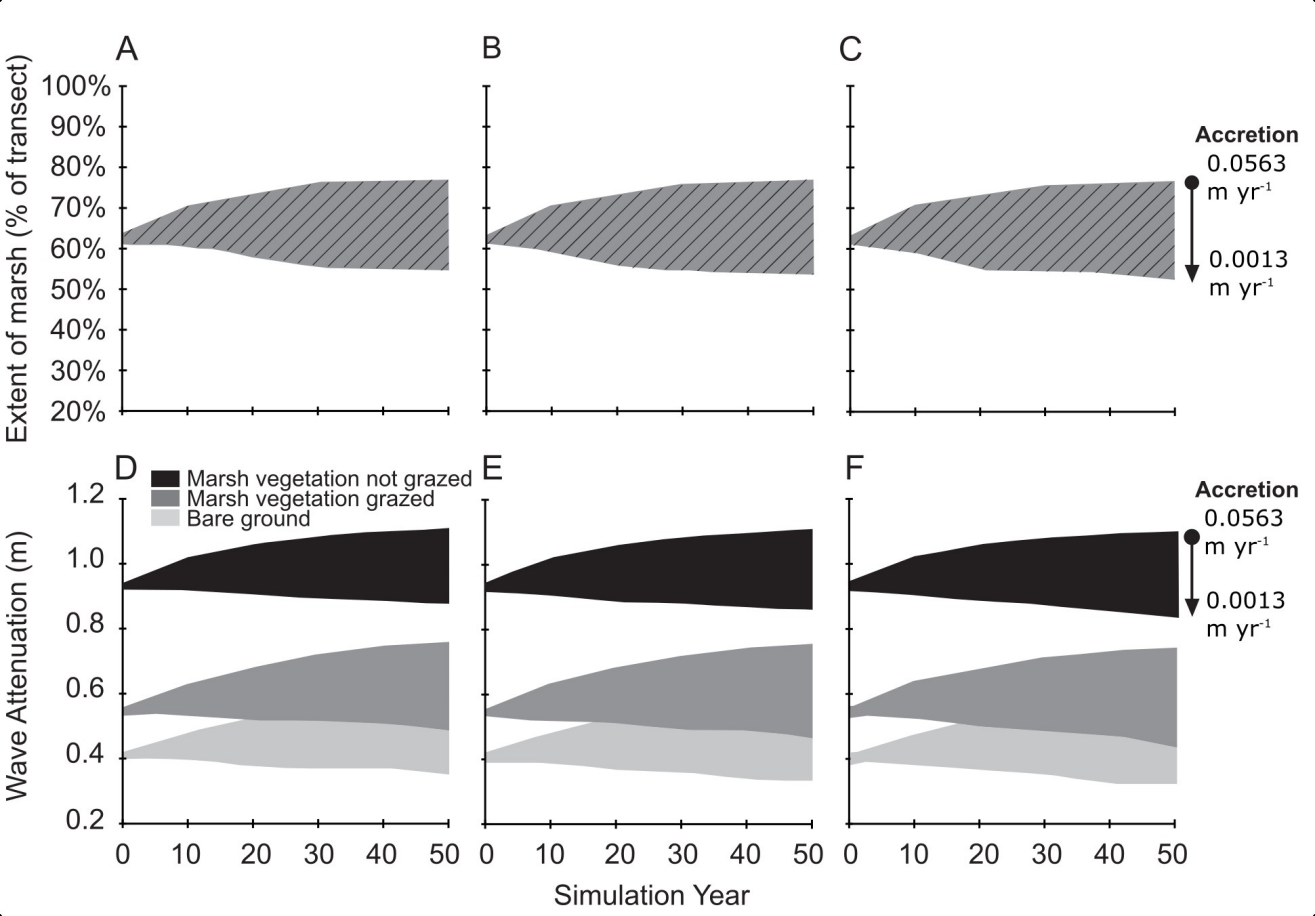
Change in wetland extent and wave attenuation in a prograding wetland (Wadden Sea) under a range of sea-level rise and accretion scenarios and management actions. Extent of marsh and wave attenuation reported as a range across the three accretion rates (Table 1) and for three sea level rise scenarios: (A,D) 0.217 m of SLR, (B,E) 0.319 m of SLR, and (C,F) 0.386 m of SLR.

The degrading and prograding sites exhibited contrasting responses to the nine future change simulations (three SLR and three sediment accretion rates) resulting in substantial differences in predicted wetland presence. In the degrading case, under all nine simulations, wetland extent decreased, with the fastest rate of decline occurring in the last ten years of simulation under the medium and high SLR scenarios (Fig 2A–2C). Percent of vegetation present was moderately stable in the prograding wetland study site, with a difference of -0.1% of vegetation from year 0 to year 50 under the lowest accretion rates and difference of +0.1% of vegetation from year 0 to year 50 under the highest accretion rates (Fig 3A–3C).

These distinct differences between the degrading and prograding wetlands translated into markedly different potential future wave attenuation, measured as the change in wave height between the start and end of the transect from XBeach. An increase in wave attenuation equates to greater reduction in wave height, while a decrease in wave attenuation equates to less reduction in wave height. The greatest increase in wave attenuation over time occurred in the prograding wetland under the lowest SLR and highest accretion rate scenario, in which attenuation of the 1.85 m incoming wave increased from 0.93 m at the start of the simulation to 1.1 m at simulation year 50 (Fig 3D). At the prograding site, even under the future scenario with greatest SLR and least sediment accretion, wave attenuation only decreased by 0.04 m over a 50-year period (Fig 3F). In contrast, wave attenuation decreased by approximately 0.20 m from simulation year zero to year 40 and an additional 0.34 m from year 40 to year 50 in the degrading study site (Fig 2F). The decline in wave attenuation over time resulted in higher waves at the end of the 3 km transect at year 50 compared to year zero, with an increase of 0.58 m (Fig 2F). Further, high accretion rates modulated decreases in wave attenuation over time (Fig 2D–2F) as wetland extent was maintained (Fig 2A–2C). In both study sites, wave attenuation of wetlands decreased over time for all scenarios where vegetation presence decreased (Fig 2 all scenarios and Fig 3D–3F at a sediment accretion rate of 0.0013 m yr^-1^).

**Fig 4:**
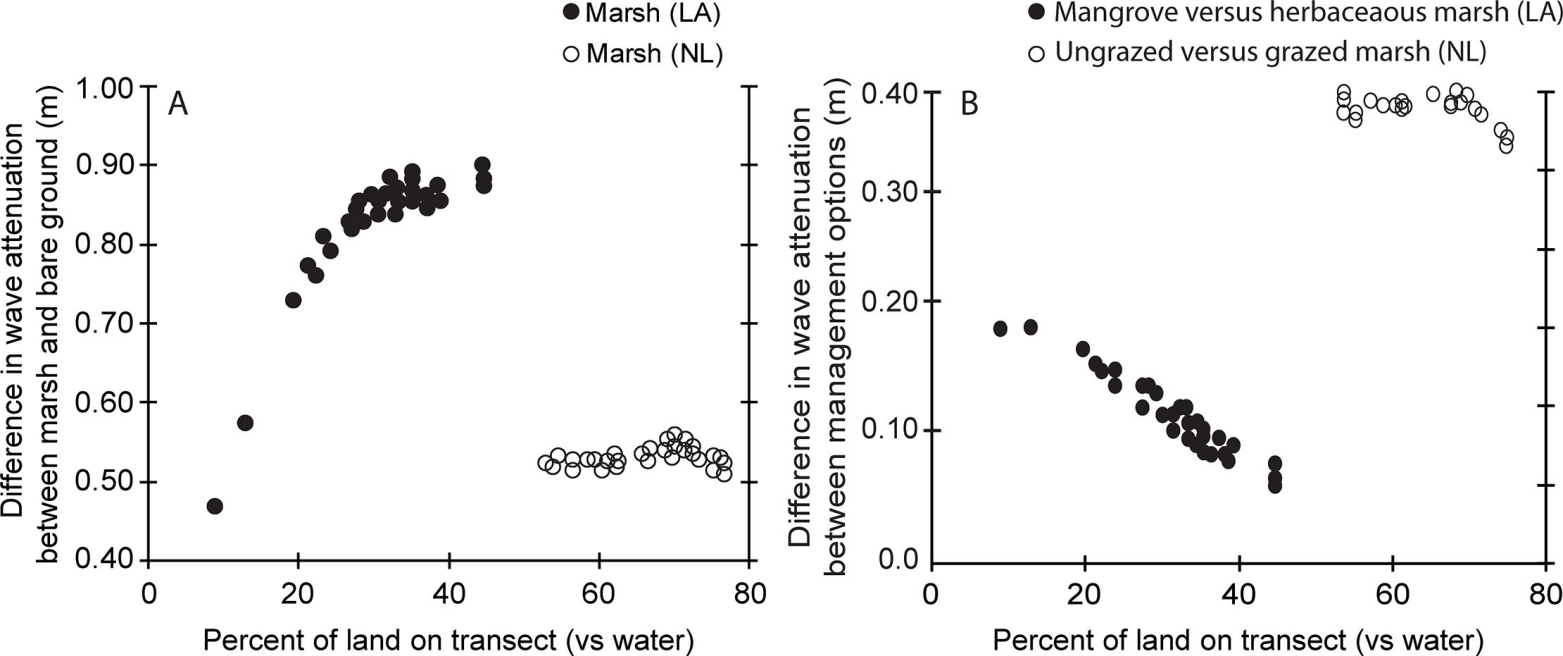
Comparison of wave attenuation across a range of land to water ratios in a degrading wetland, Louisiana, USA (LA), and an prograding wetland, Netherlands (NL). A: vegetated wetland versus bare ground, B: between management actions.

Over all future change scenarios and time points, wave attenuation was greater in the simulations with vegetation versus simulations with only bare ground, but the magnitude of that difference varied relative to the percent cover across the transect (i.e., wetland extent; Fig 4A). While herbaceous vegetation in the degrading wetland resulted in up to 0.9 m of additional wave attenuation over the modeled transect, the non-grazed wetland in the prograding wetland resulted in 0.5 m additional wave attenuation over the modeled transect (Fig 4A). Below approximately 25% land (75% water) along the transect, the additional wave attenuation benefit of vegetation rapidly declined; this was only seen in the degrading wetland as the prograding wetland was at least 50% land in all modeled scenarios (Fig 4A). The difference in wave attenuation between vegetation and bare ground simulations increased as wetland extent increased to approximately 30% of the transect, and then leveled off (Fig 4A). An increase in vegetation cover on the transect beyond 30% did not considerably increase wave attenuation relative to having only bare ground on the transect.

### Effects of management actions on future wave attenuation

In the degrading wetland (Louisiana), the replacement of herbaceous vegetation with mangroves in the model resulted in additional wave attenuation across all SLR and accretion scenarios (Fig 2). The greatest difference in wave attenuation between simulations with mangrove characteristics (new management action) and herbaceous characteristics (traditional management) was observed when the percent of transect with vegetation was lowest (Fig 4B). In other words, the additional wave attenuation benefit of the mangrove management action over the traditional management action declined as the percent cover increased on the transect (Fig 4B), or mangroves provide relatively larger benefits for narrow wetlands. In the prograding wetland (NL), the analysis to test the effects of adding grazing pressure on the wetland vegetation, showed a decline in wave attenuation of 40% compared to the un-grazed simulations. This represents a predicted increase in wave height (reduction in wave attenuation) of approximately 0.4 m across all scenarios (Figs 3 and 4B). The non-grazed management action in the prograding wetland (NL) resulted in 0.4 m additional wave attenuation over the transect relative to the grazed management action (Fig 4B). In the prograding wetland, the difference between grazed and un-grazed vegetation influence on wave attenuation lessened once the percentage of land was greater than 70% (30% water) (Fig 4B).

## Discussion

The use of two, linked models serves as an illustrative approach for exploring ecosystem service provisions over decadal time scales and for informing coastal flood risk management. Model results indicate non-linear effects of environmental conditions and management actions on wave attenuation potential of coastal wetlands in two study sites. While this study focused on the exploration of the effects of climate scenarios, environmental uncertainties, and management decisions, the approach could also be used to explicitly evaluate tradeoffs in management objectives (e.g., biodiversity vs. risk reduction), explore how wave dissipation varies for different storm wave scenarios, and identify critical change or tipping points in the system where ecosystem service provisions may be lost entirely. The model approach utilized here may be useful to managers confronted by complex problems, who often have to rely on conceptual modeling approached [96] or qualitative weight of evidence approach [97] when process-based numerical models of complex systems are not available.

Despite the simplified approach, the SLAMM results are consistent with previous studies for coastal Louisiana where historically high rates of wetland fragmentation and conversion to open water are prevalent in the study area modeled [54,98,99]. In the Wadden Sea case study used here, SLAMM results indicate wetland extent will be fairly stable under multiple sea-level rise and accretion scenarios. Similarly, an earlier study predicted marsh elevation to keep pace with sea-level rise over 100 year time period [100]. Others have noted the importance of maintaining a sufficient sediment supply in the Wadden Sea in order to sustain the marshes under sea-level rise [101], through management intervention if necessary [102].

Several assumptions made in this study, e.g., the sea-level rise rates and modelling accretion rate as equivalent to elevation change, impact the frequency, duration, and magnitude of wetland flooding and thus wetland submergence and conversion to open water. Furthermore, additional processes, such as the generation of sediment by wave-induced erosion on the marsh edge and the movement of sediment through marsh creek systems could change accretion patterns. Future work using more process-based models that incorporate dynamic linkage between sediment deposition, vegetation, accretion, and elevation change could provide greater insight but presently such detailed models are too complex, costly and time consuming for many management applications.

The analysis also demonstrates that the effect of vegetation type on wave attenuation varies as wetland landscapes change over time. In the Louisiana site, as vegetation converted to open water over time, establishing mangroves provided 0.05 m to 0.18 m (Fig 4B) more wave attenuation than herbaceous wetland vegetation. However, rates of wave attenuation were predicted to change non-linearly over time in the degrading wetland, with a steady decline in wave attenuation to year 30 and a rapidly decreasing rate after year 40, indicating potential critical change points in wave attenuation with reduction in wetland extent along the transect. The extent of marsh on the transect decreases markedly in the low accretion scenario around year 30, likely indicating that the initial ‘elevation capital’ of the marsh has been overtaken by sea-level rise at the lower accretion rates. For both the degrading (Louisiana) and prograding (the Netherlands) cases it was demonstrated that vegetation provided sufficient additional wave attenuation (vs. no vegetation) on foreshores over time under various future scenarios of change to be relevant to management decision making. Further testing could be conducted to explore the role of other management measures such as using dredged material to fill existing open water, or the timing of such measures in order to maintain a minimum future extent of vegetation in front of the levee to provide wave attenuation under design storm conditions.

Over all future change scenarios and time points analyzed, we observed 1.09 m to 1.60 m reduction in wave height over the 3 km transects, which equates to an approximate 0.02% to 0.03% decrease in wave height per meter. Direct comparisons of these values to other studies are confounded by the fact that wave dissipation varies in response to factors such as wave heights, water depths, vegetation characteristics and extent, and distance traversed [9,56,103,104]. For instance, previous studies [10] observed 20% dissipation in non-breaking waves over a 40m distance (i.e., 0.5% per m), while others [3] report < 0.025% dissipation per m at distances >1,000 m. Moreover, our study did not assume 100% coverage of vegetation across the entire transect, and instead we parameterized the model taking into account current and future wetland extent. Thus, it is important to note that the results here provide an indication of wave attenuation potential over a range of specific simulated environmental and management conditions.

In cases where wetland extent was less than 40% in comparison to open water, presence of *A*. *germinans* instead of a dominant herbaceous species, was found to potentially double wave attenuation (Fig 4B). The location modeled was a brackish and saline wetland that has experienced high fragmentation since 1985 [54], suggesting the need for restoration action. For wetland restoration efforts with wave attenuation as a primary goal, including mangrove *A*. *germinans* as a component of vegetation plantings has potential to increase realized wave attenuation. *A*. *germinans* is a native, but currently transitional, species in coastal Louisiana, and the use of a transitional species in coastal restoration poses the potential risk that planted or established mangroves may still be lost in a very cold winter, even though the frequency of these extreme cold events is predicted to decline [58]. Therefore, combinations of herbaceous wetland vegetation and mangroves may provide the most resilience in the wave attenuation function, supporting the general conclusion that having a range of species can stabilize ecosystem processes in response to variation and disturbance [105].

Within the prograding wetlands of the Dutch Wadden Sea, the practice of grazing to enhance the ecological diversity of foreshores has the potential to almost eliminate the added wave attenuation function provided by vegetation. Stable and high sediment supply was found to result in minimal changes in wave attenuation over the next 50 years through the range of SLR and accretion scenarios. However, grazing (versus no grazing) was still predicted to result in approximately 0.4 m reduction in wave attenuation (Fig 4B). As the overall system was predicted to be stable, managers would need to decide whether this reduction in wave attenuation was sufficiently beneficial to eliminate grazing. Rotated grazing regimes with intermediate grazing pressure have been shown to benefit plant diversity [60], and results of this research suggest that alternative grazing regimes may also increase the wave attenuation function provided by wetlands in these prograding wetlands depending on the effect on vegetation structure. Some studies have also shown that grazing by large herbivores, such as cattle and horses, can reduce accretion rates as a result of trampling [83], while there is no effect of small herbivores (hares and geese). However, other studies have shown that stocking densities and the activity of large herbivores affects accretion [106]. The current study only considers the effect of grazing on vegetation height; further analysis would be needed to examine the combined effect of changes in vegetation structure and accretion. However, given the status of the Wadden Sea marshes examined in this study (prograding, high sediment supply) any effect of grazing on future wave attenuation may be more through changes to vegetation rather than relative elevation.

Increased understanding of design, maintenance, management, and governance is required for successful management of natural ecosystems for wave attenuation and shoreline protection [107]. For both a degrading and prograding wetland system the combination of SLAMM and XBeach models provided insight on how management decisions and wetland structure could influence future wave attenuation.

## Conclusion

Planning for effective disaster risk reduction in coastal areas requires predictions of wave attenuation that considers future sedimentation, geomorphic configuration and vegetation cover. Specifically, incorporation of local landscape context and uncertainty regarding predicted rates of sediment accretion, subsidence, and local eustatic sea-level rise is essential. This study has shown the utility of relatively simple models to evaluate potential future landscapes that can be used to estimate hydraulic loading conditions (e.g., wave height) on levees and inform risk reduction planning.

Management decisions and actions related to coastal vegetation type and structure have the potential to change future coastal wave attenuation at a spatial scale relevant to coastal protection planning. It is increasingly well documented that coastal vegetation provides wave attenuation and planning models for disaster risk management at small and medium spatial scales could benefit from considering a range of potential future vegetation conditions, including how management related vegetation changes can contribute to or detract from risk reduction needs.

The complexities of future changes in natural systems can be daunting for coastal managers to consider and biogeomorphic contributions to coastal disaster risk reduction may not be routinely considered. This study has demonstrated how a large number of possible future scenarios can be readily analysed using freely available software that simplify complex interactions. In addition, it provided examples of how this comparative information could inform risk reduction planning. Such analyses could be expanded to consider different landscape configurations, vegetative conditions, and changing predictions of future sea-level rise, or further linked to models that consider the effects on other ecosystem functions and services (e.g., nursery habitat for commercially important fish species). Detailed design of risk reduction actions requires the use of more complex models that consider processes not captured in the approach used here (e.g., oblique wave attack). However, simpler approaches can be used to both expand the utility and target the use of those analyses to consider factors such as vegetative change and detailed hydrodynamics.
